# Investigating the Impact of Cu^2+^ Doping on the Morphological, Structural, Optical, and Electrical Properties of CoFe_2_O_4_ Nanoparticles for Use in Electrical Devices

**DOI:** 10.3390/ma15103502

**Published:** 2022-05-13

**Authors:** Shahroz Saleem, Muhammad Irfan, Muhammad Yasin Naz, Shazia Shukrullah, Muhammad Adnan Munir, Muhammad Ayyaz, Abdullah Saeed Alwadie, Stanislaw Legutko, Jana Petrů, Saifur Rahman

**Affiliations:** 1 Department of Physics, University of Agriculture Faisalabad, 38040, Pakistan; zshukrullah@gmail.com (S.S.); adnanmunir911@gmail.com (M.A.M.); muhammadayyaz333@yahoo.com (M.A.); 2 Electrical Engineering Department, College of Engineering, Najran University Saudi Arabia, Najran 11001, Saudi Arabia; miditta@nu.edu.sa (M.I.); asalwadie@nu.edu.sa (A.S.A.); srrahman@nu.edu.sa (S.R.); 3 Faculty of Mechanical Engineering, Poznan University of Technology, 60-965 Poznan, Poland; stanislaw.legutko@put.poznan.pl; 4 Faculty of Mechanical Engineering, Department of Machining, Assembly and Engineering Metrology, VSB—Technical University of Ostrava, 17. Listopadu 2172/15, 708 00 Ostrava, Poruba, Czech Republic; jana.petru@vsb.cz

**Keywords:** spinel ferrites, CoFe_2_O_4_ nanoparticles, Cu^2+^ doping, electrical properties

## Abstract

This study investigated the production of Cu^2+^-doped CoFe_2_O_4_ nanoparticles (CFO NPs) using a facile sol−gel technique. The impact of Cu^2+^ doping on the lattice parameters, morphology, optical properties, and electrical properties of CFO NPs was investigated for applications in electrical devices. The XRD analysis revealed the formation of spinel-phased crystalline structures of the specimens with no impurity phases. The average grain size, lattice constant, cell volume, and porosity were measured in the range of 4.55–7.07 nm, 8.1770–8.1097 Å, 546.7414–533.3525 Å^3^, and 8.77–6.93%, respectively. The SEM analysis revealed a change in morphology of the specimens with a rise in Cu^2+^ content. The particles started gaining a defined shape and size with a rise in Cu^2+^ doping. The Cu_0.12_Co_0.88_Fe_2_O_4_ NPs revealed clear grain boundaries with the least agglomeration. The energy band gap declined from 3.98 eV to 3.21 eV with a shift in Cu^2+^ concentration from 0.4 to 0.12. The electrical studies showed that doping a trace amount of Cu^2+^ improved the electrical properties of the CFO NPs without producing any structural distortions. The conductivity of the Cu^2+^-doped CFO NPs increased from 6.66 × 10^−10^ to 5.26 × 10^−6^ ℧ cm^−1^ with a rise in Cu^2+^ concentration. The improved structural and electrical characteristics of the prepared Cu^2+^-doped CFO NPs made them a suitable candidate for electrical devices, diodes, and sensor technology applications.

## 1. Introduction

Magnetic nanomaterials have found applications in many cutting-edge technologies, including engineering, energy, drug delivery, medical diagnostics, defense, electromagnetics, computing devices, resonance imaging, high-density magnetic storage devices, biological functions, electronic sensors, lithium-ion batteries, and microwave and data storage devices [1,2]. Magnetic materials are well-known for the aforementioned applications because of their low production cost, higher efficiency than other alloys, and outstanding characteristics at a nanoscale [3,4]. For this purpose, application-specific electrical, structural, morphological, optical properties, as well as several other properties of ferrites, have been studied over time. The addition of divalent and trivalent ions to the tetrahedral (A) and octahedral (B) sites of the spinel ferrites’ lattice can control these properties [5,6]. Cobalt ferrite (CFO) is a hard ferrite, which piqued the interest of researchers because of its strong crystallographic anisotropy, high Curie temperature, high coercivity, and large saturation magnetization. At ambient temperature, cobalt ferrites have an inverse spinel structure with a high degree of inversion and exhibit ferrimagnetism, with Fe^3+^ ions evenly distributed among the tetrahedral A-sites and octahedral B-sites, and Co^2+^ ions occupying the octahedral B-sites [7].

The magnetic, electric, and opto-physical characteristics of CoFe_2_O_4_ can be tuned using different metal additives like Ce, Al, Ga, In, Zn, and Cr. These properties, combined with their high physical and chemical stability, make CFO NPs suitable for magnetic fluids, data storage, lithium-ion batteries, magnetic recording, targeted drug delivery, catalysts, hyperthermia, and biosensors. In other words, CFO has applications in both magnetic and electrical fields. Cobalt ferrites’ electrical and magnetic properties can be modified by adding small amounts of impurities, thus allowing them to be used in various applications. The cations of transition metals are easy to incorporate into the lattice of a magnetite structure. During the past few years, several research investigations have been carried out to study the different parameters of pure and doped CFO NPs. Jabbar et al. [8] inspected the impact of different concentrations of Mn^2+^ on the structural and dielectric attributes of CFO NPs. An increase in average crystallite size was observed from 10.79 nm to 14.28 nm with a rise in Mn^2+^ concentration from 0.2 to 0.6, and was then reduced to 9.95 nm with a further rise in the concentration of Mn^2+^ to 0.8. The dielectric characteristics of the synthesized specimens were reduced by an increment in the doping rate of Mn^2+^. Hysteresis loops indicated a dramatic decline in saturation magnetization when the doping ratio was increased. Kamran et al. [9] synthesized cerium-doped cobalt ferrite for resistive RAM applications. Cobalt ferrites with different Ce concentrations (*x* = 0.1, 0.2, 0.3, 0.4, and 0.5) were produced using the coprecipitation approach. The effect of Ce concentration on the switching, dielectric, and electric characteristics were probed. The XRD analysis indicated the formation of a spinel structure and revealed the formation of a secondary phase of CeO_2_. The dielectric loss, AC electrical conductivity, and dielectric constant were diminished with an increase in Ce content. The I–V curves demonstrated a hysteresis loop nature by confirming the existence of a resistive switching effect in Ce-incorporated CFO. The research outcomes recommended that Ce-doped cobalt ferrites are a strong contender for resistive RAM applications.

Jesus et al. [10] conducted comprehensive research on the effect of Mg^2+^ doping on the magnetic, electric, and thermal features of CFO. Co_1−*x*_Mg*_x_*Fe_2_O_4_ (*x* = 0, 0.08, 0.16, and 0.24) was successfully synthesized via the sol−gel auto-ignition route. The XRD analysis confirmed the formation of cubic spinel ferrite with a reduction in lattice parameters when the Mg^2+^ content was increased. The FESEM investigation showed a uniform grain distribution with a spherical particle shape. The obtained FTIR spectra with two prominent bands endorsed the formation of spinel ferrites, as mentioned in the XRD spectra. The DC electrical resistivity also improved with an increase in Mg^2+^ content in the specimen. It was concluded that doping showed a significant impact on the magnetic, dielectric, and physical characteristics of the cobalt ferrites. It was also revealed that the doping of Cu^2+^ to CFO caused Co^2+^ ions to shift from octahedral to tetrahedral sites, resulting in structural modifications, which made it a highly suitable material for the preparation of catalysts, sensors, and electrical devices [11]. Electrical conductivity was also greatly increased in another investigation linked to the impact of Cu^2+^ doping on several properties of cobalt ferrites. Because of their mild conductivity, Cu^2+^-doped spinel ferrites have previously been shown to have excellent properties for possible use in sensors, electronic devices, and catalysis [12]. In the current study, Cu^2+^ was chosen for doping in CFO because it plays a crucial role in enhancing the magnetic and electrical properties of the spinel ferrites.

It is common knowledge that synthesis methods, precursor chemical composition, sintering time, and temperature significantly impact the quality and characteristics of spinel-ferrite materials. Different approaches are used to produce CFO NPs, including co-precipitation [10], spray pyrolysis [13,14], microemulsion [13], facile sol–gel, sol−gel auto combustion [15], hydrometallurgical processes [16], complexometric methods [17], hydrothermal methods [18,19], and microwave method [18]. One of the key challenges with the co-precipitation approach is that achieving optimal particle size control throughout the synthesis process is difficult [20]. Ceramic technology is simple to implement in the industry, but it consumes a lot of energy. At an industrial level, hydrometallurgical processes are prohibitively expensive or difficult to implement. With its simplicity, high product purity, higher control ratio, and more uniform product composition, the sol−gel technique is assumed to be the more suitable method among various preparation methods [21]. Thus, this paper reports the synthesis of Cu^2+^-doped CFO spinel ferrites via the sol−gel method. We studied the effects of Cu^2+^ doping on the morphological, structural, optical, and electrical properties of CFO NPs for electrical applications.

## 2. Materials and Methods

Numerous studies [8,9,22,23] have been conducted on smaller amounts of Cu doping, where an increase in Cu^2+^ concentration resulted in structural modifications with increased electrical characteristics and decreased magnetic features of materials. Jnaneshwara et al. [24] fabricated Co_1−*x*_Cu*_x_*Fe_2_O_4_ with *x* = 0 to 0.5 using an auto-combustion route and revealed a decrease in the magnetization saturation value from 38.5 to 26.7 emu/g with an increase in Cu^2+^ concentration. Ghosh et al. [22] prepared Cu*_x_*Co_1−*x*_Fe_2_O_4_ with (*x* = 0.00, 0.15, 0.30, 0.45, and 0.60) by employing a chemical co-precipitation route. The substitution of Cu^2+^ ions in CFO NPs decreased the hopping length and boosted the electrical conduction process. This shows that the dopant concentration and synthesis technique influenced the size and morphology of the magnetic nanomaterials, which, in turn, influenced their electrical and magnetic characteristics. Therefore, in this research work, the desired composition of Cu_1−*x*_Co*_x_*Fe_2_O_4_ was chosen with a higher dopant (Cu^2+^) ratio of *x* = 0.04, 0.08, and 0.12.

A sol–gel technique was applied to synthesize the nanocrystalline copper-doped cobalt ferrite (Cu_1−*x*_Co*_x_*Fe_2_O_4_) with a composition of x = 0.04, 0.08, and 0.12. In a typical process, stoichiometric amounts of cupric chloride (CuCl_2_.2H_2_O), cobaltous chloride (CoCl_2_.6H_2_O), and anhydrous ferric chloride (FeCl_3_) were dissolved separately in distilled water to obtain homogenous solutions. The prepared solutions were magnetically stirred for 20 min to complete the homogeneity. The prepared solutions were intermixed under continuous stirring. The neutralization mechanism was attained by ammonia addition into the solution with the pH maintained between 7 and 8. After continuous stirring and heating for 5 h, the ammonia-added solution turned into a transparent gel, which was converted into a viscous gel upon further heating. The obtained gel was furnaced for 6 h at 500 °C to obtain burnt ash. The ash powder was ground to obtain fine powdered Cu^2+^-doped CFO NPs.

The as-produced CFO NPs were examined through an X-ray diffractometer (MSAL-XD2, Cu-Kα radiation) for the structural analysis, scanning electronic microscope (SEM) (HITACHI S-520) for the morphological analysis, a UV−VIS spectrometer (Perkin Lambda 25) for the optical absorption spectra analysis, and a Keithley Electrometer (Model: 2410-C) for the I–V characteristic curve analysis. The liquid suspension was prepared by mixing 10 mg of sample in 5 mL of distilled water for the UV−VIS spectral analysis. Pellets of 3 mm thickness were grown by applying a pressure of 3 tons in a hydraulic press machine to study the I–V characteristics of the prepared ferrite nanoparticles.

## 3. XRD Analysis

**Figure 1:**
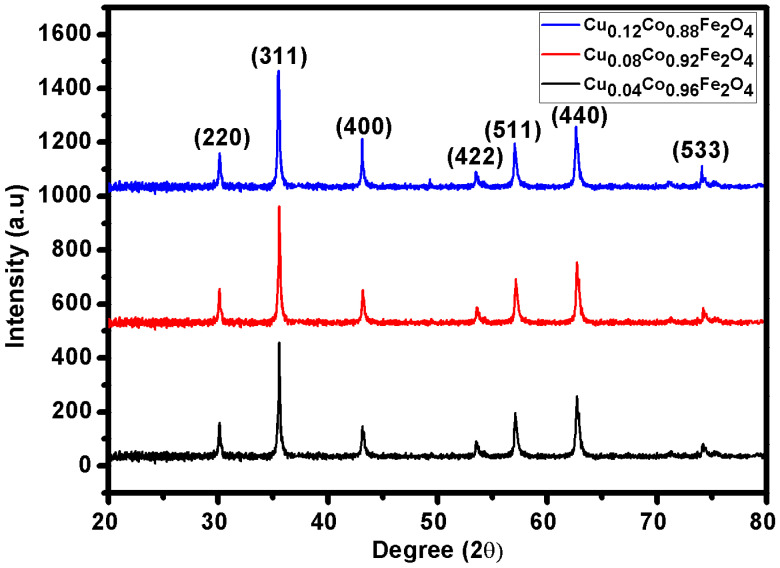
XRD patterns of CFO NPs doped with Cu^2+^ ions.

The powdered Cu^2+^-doped CFO NP samples were investigated for phase identification and various structural characteristics by generating XRD spectra with an MSAL-XD2 diffractometer (*λ* = 1.54 Å) in a 2θ range of 20–80° and at a scanning rate of 2°/min. Figure 1 reports the XRD spectra of the CFO NPs doped with different Cu^2+^ concentrations. The obtained XRD patterns revealed the pure crystalline nature of the prepared Cu^2+^-doped CFO NPs with no secondary/impurity phases, which confirmed the high purity of the spinel-phase cubic structure of the product. The intensity of the peaks increased with the increasing Cu^2+^ content, as seen from the characteristic peak (311) in Figure 1. This increase may be from the structural modifications caused by the Cu^2+^ ions in the CFO structure. However, no peak related to oxides of copper, metallic copper, or any binary cobalt−copper phase was seen in the pattern. This was possibly because of the smaller intensities of copper oxide peaks compared with copper ferrites, which were difficult to distinguish. Therefore, Cu^2+^ substitution in CFO sites may have improved its structural features without any lattice distortions or imperfections. The comparable ionic radii of Cu^2+^ (0.73 Å) and Co^2+^ (0.70 Å) may be another cause for the unchanged lattice structure and high crystallinity.

The average grain size (*D*) of the Cu^2+^-doped CFO NP samples was obtained using Debye−Scherrer’s formula, given below in Equation (1) [7].

(1) $$D=\frac{K\lambda }{\beta \cos\theta }\;$$

In this equation, *K* is a constant (0.9), which is also called the shape factor; *β* is the full-width half maxima; *θ* is Bragg’s diffraction angle; and *λ* is the wavelength of the X-rays (1.54 Å). The average crystallite size increased from 4.55 nm to 7.07 nm as the Cu^2+^ content increased in the specimen, as summarized in Table 1.

A decrease in the broadness of XRD reflections was due to the increased crystallite size. The peak intensities increased in return because of the reduced broadness. The unit cell parameter of the Cu^2+^-doped CFO NPs linearly decreased when increasing the Cu^2+^ concentration in the composition, which obeyed Vergard’s law. Thus, the decreasing trend in the lattice parameter was caused by the large ionic radius of Cu^2+^ (0.73 Å), which replaced the small ionic radius sites of Co^2+^ and/or Fe^3+^ (0.70 Å/0.64 Å) ions in the host structure. A decrease in cell volume of the crystal structure with the Cu^2+^ content was because of the reduction in the lattice constant. Thus, substituting relatively larger ionic radii Cu^2+^ at the sites of the relatively smaller ionic radii Co^2+^/Fe^3+^ resulted in a decrease in lattice parameters from 8.1770 Å to 8.1097 Å and cell volume from 546.7414 to 533.3525 Å^3^. The lattice parameters, including the lattice constant (*a*) and cell volume (*V*_cell_), were calculated using the following relations [4,25]:

(2) $$a=d\sqrt{h^{2}+l^{2}+k^{2}}$$

(3) $$V=a^{3}$$

The calculated X-ray density value decreased from 2.851 g/cm^3^ to 2.742 g/cm^3^, as given in Table 1. It is revealed that the X-ray density decreased as a result of a continuous decrease in the molar mass of the sample. The larger atomic weight of the substituted Cu^2+^ ions compared with the Co^2+^ ions is another reason for the decrease in X-ray density. The X-ray density (*ρ_X_*) was obtained from Equation (4) [26]:

(4) $$\rho_{X}=\frac{8\;M}{N_{A}a^{3}}\;$$

where *a* is the lattice constant, *M* is the molar mass, and *N_A_* is the Avogadro’s number. The bulk density $\rho_{B}$ was calculated from Equation (5). The bulk density value decreased from 2.601 g/cm^3^ to 2.553 g/cm^3^ with an increase in Cu^2+^ concentration. The pores that formed during the preparation procedure were responsible for the lower bulk density. The bulk density was calculated using the following formula [27]:

(5) $$\rho_{B}=\frac{m}{V}$$

where *m* is the mass and *v* is the volume. The values of the X-ray density and bulk density were used to calculate the percentage porosity. Table 1 shows the change in porosity with Cu^2+^ concentration in the sample. The X-ray density, bulk density, and porosity of the CFO NPs decreased with an increase in Cu^2+^ concentration. Typically, porosity is defined as the ratio of the pore volume and total volume of the particles. Porosity provides essential information about the morphology and optical properties of materials. Porous materials offer a larger surface area to absorb a large number of molecules as well as lower density or light weightiness of material. Moreover, the size of the pores can also be used as a sieve for the separation of molecules. The lowest value of porosity corresponds to the highly-dense structure of the synthesized ferrites. The porosity of Cu^2+^-doped CFO NP samples was calculated using the following formula [28]:

(6) $$Porosity=\left(1-\frac{\rho_{B}}{\rho_{X}}\right)\times 100\%$$

where $\rho_{B}$ denotes the bulk density and $\rho_{X}$ is the X-ray density. The percentage porosity was calculated from Equation (6) by placing $\rho_{X}$ and $\rho_{B}$ from Equations (4) and (5) into Equation (6), respectively.

## 4. Dislocation Density

The dislocation density (*δ*_hkl_) measures the length of the dislocation lines per unit volume and indicates the number of defects in the crystal structure. The dislocation density of the prepared Cu_1−*x*_Co*_x_*Fe_2_O_4_ ferrites increased with an increase in Cu^2+^ concentration, as summarized in Table 2. The dislocation density of the prepared ferrites shows a direct relation with the growth in crystallite size and an inverse relation with the lattice constant. The increase in *δ*_hkl_ upon increasing the Cu^2+^ content might be because of the movement of Cu atoms from the grain boundary to the grain sites. The variation in dislocation density strongly affects the structural parameters of the nanomaterials. The presence of dislocations shows that the dopant elements perfectly replace the host ions in the crystal structure. Therefore, very small crystal defects are produced, and the crystal structure of the CFO ferrites is modified/improved. The dislocation density (*δ*_hkl_) was calculated using Equation (7) considering the crystallite size, lattice constant, and strain data [29]:

(7) δhkl=15εa.D

where *D* is the crystallite size, *a* is the lattice constant, and *ε* is the strain.

## 5. Microstrain

Williamson−Hall (W−H) plots were produced to investigate the impact of the crystallite size and microstrain on peak broadening [30].

(8) $$\beta_{hkl}\cos\theta =\frac{Κ\lambda }{D}+4\varepsilon \sin\theta$$

The following equation was used to calculate the microstrain [31].

**Figure 2:**
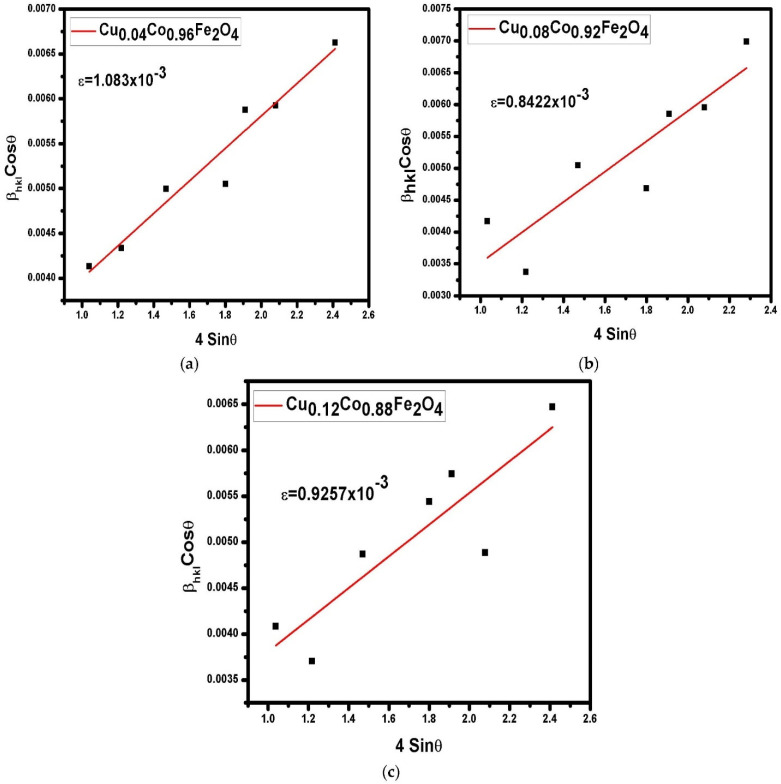
W–H plots of CFO NPs doped with different concentrations of Cu^2+^ ions. (**a**) Cu_0.04_Co_0.96_Fe_2_O_4_, (**b**) Cu_0.08_Co_0.92_Fe_2_O_4_, (**c**) Cu_0.12_Co_0.88_Fe_2_O_4_.

(9) ε=β/4tanθ

where *K* is the geometry-dependent constant (0.9 for spherical nanoparticles), *D* is the mean grain size, *θ* is the Bragg’s angle, *λ* is the wavelength of the X-rays, and ε represents the microstrain. The W−H plots of the CFO NP specimens are reported in Figure 2. The obtained values for the microstrain and crystallite size are given in Table 2. The microstrain values decreased from 1.08 × 10^−3^ to 0.925 × 10^−3^ with a reduction in grain size from 7.07 nm to 4.55 nm. As seen by the decreasing slope of all W−H graphs, the detected microstrains in the synthesized samples were significant in nature. The slope of the W−H plots increased with the increase in Cu^+2^ content in the sample, as shown in Figure 2. In CFO ferrite, almost all Co^2+^ ions occupy octahedral sites. Substituting the Cu^2+^ dopant into the CFO ferrites may result in a strong migration possibility of a slight proportion of Cu^2+^ ions towards the tetrahedral sites. Thus, the substitution of Cu^2+^ dopant into CFO ferrites may result in a significant increase in the microstrain of CFO NPs.

## 6. Scanning Electron Microscopy

**Figure 3:**
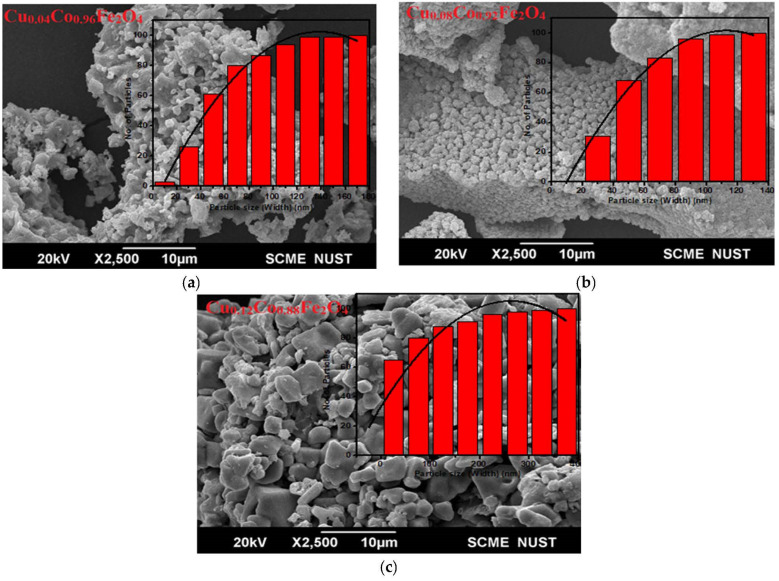
SEM images of (**a**) Cu_0.04_Co_0.96_Fe_2_O_4_ (**b**) Cu_0.08_Co_0.0.92_Fe_2_O_4_, and (**c**) Cu_0.12_Co_0.88_Fe_2_O_4_ nanoparticles.

The SEM micrographs were produced to reveal the morphology and approximate the particle size of the synthesized Cu^2+^-doped CFO NPs. SEM images, as shown in Figure 3a–c. In our case, dopant (Cu^2+^) concentration has shown a sufficient effect on the size and morphology of the synthesized nanoparticles. Figure 2a–c shows 20 μm scale SEM images of the synthesized samples. The CFO samples, doped with 4%, 8%, and 12% concentrations of Cu^2+^, revealed average particle sizes of 44 nm, 45 nm, and 48 nm, respectively. Figure 3a shows a sponge-like structure with a lot of aggregation and porosity, which might be because of the immiscible nature of copper and cobalt at this concentration. The grains were distributed nonuniformly with different cluster/agglomerate-type structures. As a result of different shapes or structures, the grain boundaries were not clear in the case of the Cu_0.04_Co_0.96_Fe_2_O_4_ sample and the porosity was measured to be about 8.77%, which shows that the maximum number of pores were formed. Figure 3b shows that CFO NPs doped with 8% of Cu^2+^ were also nonuniformly distributed with a high agglomeration. The formation of agglomerated or cluster-like structures led to the formation of unclear grain boundaries. Because of the interparticle interactions caused by the dipole−dipole interaction and Van der Waals force, very few nanoparticles merged into larger nanoparticles, resulting in moderate agglomeration [22]. Many pores on the surface of particles were observed with several layers of nanoparticles. The porosity of 8% Cu^2+^-doped CFO samples was measured to be about 7.85%. Figure 3c shows that 12% of Cu^2+^-doped CFO NPs exhibited a nonuniformly distributed nanoplate-like morphology with clear grain boundaries. In contrast, the porosity value decreased by up to 6.93%, which was the minimum value observed among all of the samples.

## 7. UV Analysis

**Figure 4:**
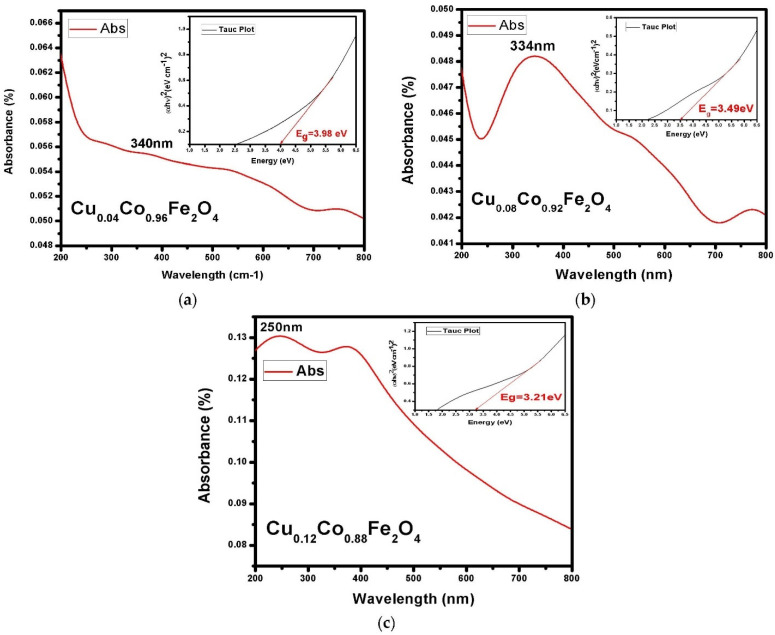
UV−VIS absorption patterns of Cu^2+^ of CFO NPs doped with different concentrations of Cu^2+^ ions. (**a**) Cu_0.04_Co_0.96_Fe_2_O_4_, (**b**) Cu_0.08_Co_0.92_Fe_2_O_4_, (**c**) Cu_0.12_Co_0.88_Fe_2_O_4_.

UV−VIS spectroscopy analysis was carried out to investigate the optical response of Cu^2+^-doped CFO NPs. The absorption spectra and their corresponding band gap Tauc-plots are shown in Figure 4. The absorption spectra were in the photon wavelength range of 200–800 nm. The absorbance generally depended on factors such as the lattice parameters, grain size, impurity centers, surface roughness, and energy band gap [32]. Figure 4 clearly shows that the absorption spectra of the prepared ferrites were in the visible region, with absorption edges at 340 nm, 334 nm, and 250 nm for the samples of Cu_0.2_Co_0.8_Fe_2_O_4_, Cu_0.4_Co_0.6_Fe_2_O_4_, and Cu_0.6_Co_0.4_Fe_2_O_4_, respectively. The shifting of absorption edges of the spectra towards a shorter wavelength when increasing the Cu^2+^ content showed a blue shift of light. This shift in absorption edge peak resulted in variations in the optical band gap energy as a result of quantum confinement [33].

The Tauc relation was used to establish the samples’ direct band gap. The direct band gap relation was used for the calculation of the energy band gap of Cu^2+^-doped CFO NPs, as given in Equation (10) [34]:

(10) $$\left(\alpha hv\right)=k{\left(hv-E_{g}\right)}^{1/2}$$

where α is an absorption coefficient, *hν* is the energy of photon, *E_g_* is the energy band gap, and *A* is a constant. Equation (11) was used to determine the absorption coefficient *a* of the synthesized samples.

(11) $$a=\frac{2.303\times A}{t}$$

where *A* is the absorption and *t* is the sample thickness. The Tauc plots of the prepared ferrites were obtained by plotting a curve between eV and (*ahν*)^2^, as shown in Figure 4. The linear part of the curve was extrapolated to plot the slope intercept through which the band gap energy was calculated. The band gap decreased from 3.98 eV to 3.21 eV as the Cu^2+^ concentration increased. The reduction in band gap with the increase in the size of the nanoparticles could be assigned to Brass’s model. According to Brass’s model, the energy band gap and particle size are related to each other by the relation given below in Equation (12):

(12) $$E_{g}=E_{g}^{bulk}+\frac{h^{2}\pi^{2}}{2er^{2}}\left(\frac{1}{m_{e}}+\frac{1}{m_{h}}\right)-\frac{1.8e^{2}}{4\pi \varepsilon \varepsilon_{0}r}$$

where *E_g_*^*bulk*^ is the bulk energy gap; *E_g_* is the energy band gap; *r* is the particle size; *h* is equal to *h*/2π; *m_e_* is the effective mass of electrons; *m_h_* is the effective mass of holes; *e* is the electron charge; and *ε*_0_ are *ε* the permittivity of free space and relative permittivity, respectively. Equation (11) shows that *E_g_* and the particle size are inversely proportional to each other. The larger size particles exhibited a lower band gap [35]. Thus, the energy band gap of Cu^2−^ doped CFO NPs was influenced by increasing the particle size and decreasing the lattice constant. The variation in particle size with Cu^2+^ concentration is shown in Figure 4.

## 8. I–V Characteristics

**Figure 5:**
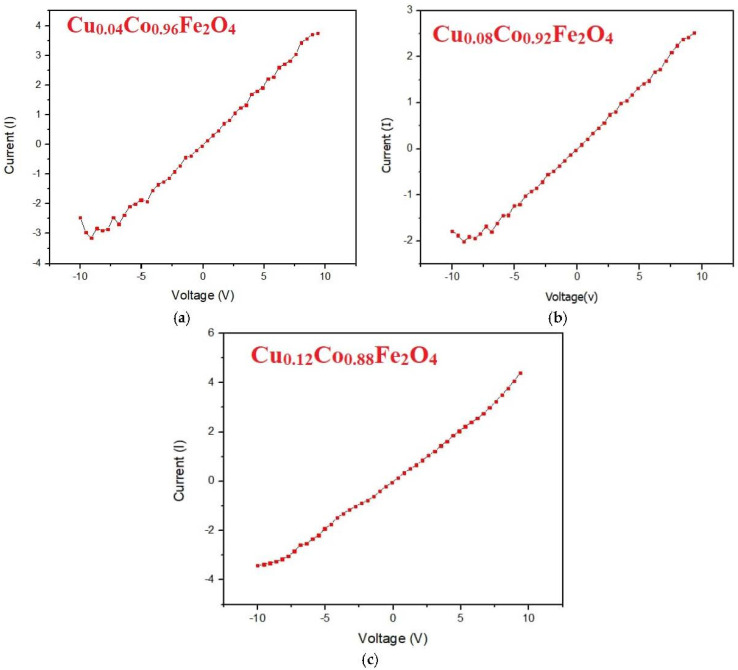
I–V characteristics of CFO NPs doped with different concentrations of Cu^2+^ ions. (**a**) Cu_0.04_Co_0.96_Fe_2_O_4_, (**b**) Cu_0.08_Co_0.92_Fe_2_O_4_, (**c**) Cu_0.12_Co_0.88_Fe_2_O_4_.

The I–V characteristics of an electric device are a set of graphic curves that indicate how the specimen performs in a circuit or electronic device. The relationship between the applied voltage and the current flowing through an electronic device is used to depict these characteristic curves. These curves are commonly used to determine and comprehend the basic properties of electronic devices, as well as to investigate their behavior inside an electronic circuit. The value of the ideality factor (*N)*, obtained from Equation (13), is used to estimate the transport current of the device [36]:

(13) $$N=\frac{q}{K_{B}T}\times \frac{dV}{dlnI_{s}}$$

where *q* is the electric charge, *T* is an absolute temperature, *K_B_* is the Boltzmann constant, and *I_s_* is the saturation current. The I–V characteristics of the doped specimens were measured using a KI/I2 electrolyte. Figure 5a–c shows an Ohmic relation at a low applied voltage (*V* < 1 V), as given by Equation (14) [37].

(14) $$I\propto V^{\alpha }$$

The parameter *α* defines the Ohmic and non-Ohmic nature of a material. In the case of Ohmic materials, *α* = 1, which is also known as the low-current-Ohmic range, and *α* > 1 in the case of non-Ohmic materials. In our case, the I–V curves between 0 and 1 V showed the Ohmic nature of the prepared Cu^2+^-doped CFO NPs. These findings revealed that, in magnetic nanomaterials, the I–V characteristics provided the most accurate interpretation of the conduction mechanism [32]. The I–V characteristics of the Cu^2+^-doped CFO NPs are depicted in Figure 5. The acquired I−V curves revealed linear characteristics, showing that Cu^2+^-doped CFO NPs were Ohmic in nature. The resistivity and conductivity of CFO NPs were calculated by Equation (15), as follows:

(15) $$\rho =\frac{RA}{L}$$

where *ρ* is the resistivity, *L* is the length of the electrodes, *R* is the resistance of the specimen, and *A* is the area (*A* = π*r*^2^) of the pellet. The inverse of resistivity gives the conductivity of the material. Figure 5a–c shows the I–V characteristics of the CFO NPs doped with wt. 4%, wt. 8%, and wt. 12% concentrations of Cu^2+^. The values of electrical resistivity and conductivity of the prepared Cu^2+^-doped CFO NPs under ambient conditions are reported in Table 3.

The electrical resistivity of ferrite nanoparticles depends on the composition and crystal structure [38,39]. The results show that the resistivity of Cu^2+^-doped CFO NPs decreased with an increase in the Cu^2+^ content, while the conductivity increased. The resistivity value decreased from 1.5 × 10^9^ to 1.9 × 10^5^ Ω-cm for the samples doped with 4%, 8%, and 12% concentrations of Cu^2+^. Similarly, another report showed the electrical resistivity of CFO NPs in the range of 6.4 × 10^5^ to 33.31 × 10^6^ Ω-cm, which justified our findings. The electrical conductivity of the prepared samples increased from 6.66 × 10^−10^ to 5.26 × 10^−6^ ℧ cm^−1^ by increasing the Cu^2+^ concentration. The reduction in the energy band gap of Cu^2+^-doped CFO NPs was also attributed to a decrease in the resistivity of the prepared ferrites. These observed properties of Cu^2+^-doped CFO NPs are useful for technological applications, including the development of electronic devices and sensor technology [40].

## 9. Conclusions

In conclusion, Cu^2+^-doped CFO NPs were produced by a sol−gel route to investigate the impact of Cu^2+^ doping on the structural, optical, morphological, and electrical features of CFO NPs. The phase confirmation and purity of the prepared Cu^2+^-doped CFO NPs were tested using XRD. The single-phase spinel structure of Cu^2+^-doped CFO NPs with an average crystallite size in the 4.55–7.07 nm range was confirmed without any impurity phase. The lattice constant, cell volume, and porosity of the Cu^2+^-doped CFO NPs decreased by increasing the Cu^+2^ concentration. A very small increase in dislocation density and strain was seen as a result of an increase in crystallite size, which demonstrated the structural improvements. The agglomeration decreased by increasing the Cu^2+^ concentration, which was responsible for the decreased porosity of the prepared specimens. The energy band gap decreased from 3.98 eV to 3.21 eV by increasing the Cu^2+^ concentration, which was in good agreement with the XRD results. The resistivity of the Cu^2+^-doped CFO NPs decreased from 1.5 × 10^9^ to 1.9 × 10^5^ by increasing the Cu^2+^ concentration, which in turn increased the conductivity from 6.66 × 10^−10^ to 5.26 × 10^−6^ ℧ cm^−1^. These findings suggest that doping a trace amount of Cu^2+^ content improves the structural and electrical features of CFO NPs and makes them a promising candidate for the development of electrical devices, as well as in diode and sensor technology. However, further changes in the Cu^2+^ concentration in CFO NPs may be applied to study its detailed impact on the optical, dielectric, catalytic, magnetic, thermoelectric, and electrical characteristics of CFO NPs.

## Figures and Tables

**Table 1:** Structural properties of CFO NPs doped with Cu^2+^ ions.

CFO NPs	Grain Size (nm)	d-Spacing	Lattice Constant	Lattice Volume (Å^3^)	X-ray Density (g/cm^3^)	Bulk Density (g/cm^3^)	Porosity (%)
Cu_0.04_Co_0.96_Fe_2_O_4_	4.55	2.4655	8.1770	546.7414	2.851	2.601	8.77
Cu_0.08_Co_0.92_Fe_2_O_4_	5.33	2.4584	8.1535	542.0411	2.803	2.582	7.85
Cu_0.12_Co_0.88_Fe_2_O_4_	7.07	2.4452	8.1097	533.3525	2.742	2.553	6.93

**Table 2:** Dislocation density, crystallite size, and microstrain of Cu^2+^-doped CFO NPs.

Samples	Crystallite Size (*D)* (nm)	Dislocation Density (m^−2^)	Strain (×10^−4^)
Cu_0.04_Co_0.96_Fe_2_O_4_	4.55	0.000280	1.083 ± 0.0045
Cu_0.08_Co_0.92_Fe_2_O_4_	5.33	0.000290	0.842 ± 0.0041
Cu_0.12_Co_0.88_Fe_2_O_4_	7.07	0.000376	0.925 ± 0.0031

**Table 3:** Electrical characteristics of Cu^2+^-doped CFO nanoparticles.

Sample ID	Resistivity (*ρ*) (Ω cm)	Conductivity (σ) (℧ cm^−1^)
Cu_0.04_Co_0.96_Fe_2_O_4_	1.5 × 10^9^	6.66 × 10^−10^
Cu_0.08_Co_0.92_Fe_2_O_4_	1.7 × 10^7^	5.88 × 10^−8^
Cu_0.12_Co_0.8_Fe_2_O_4_	1.9 × 10^5^	5.26 × 10^−6^

## Data Availability

The reported data will be available from the authors upon a reasonable request.
